# Quantitative and Compositional Study of Monospecies Biofilms of Spoilage Microorganisms in the Meat Industry and Their Interaction in the Development of Multispecies Biofilms

**DOI:** 10.3390/microorganisms7120655

**Published:** 2019-12-05

**Authors:** Carolina Ripolles-Avila, Nerea García-Hernández, Brayan H. Cervantes-Huamán, Tina Mazaheri, José Juan Rodríguez-Jerez

**Affiliations:** Area of Human Nutrition and Food Science, Department of Food and Animal Science, Universitat Autònoma de Barcelona, 08193 Barcelona, Spain; carolina.ripolles@uab.cat (C.R.-A.); nereagarher@gmail.com (N.G.-H.); brayancervanteshuaman@gmail.com (B.H.C.-H.); tinamazaheri71@gmail.com (T.M.)

**Keywords:** spoilage bacteria, biofilms, ecological interactions, multispecies, control, food contact surfaces

## Abstract

Food spoilage is a serious problem in the food industry, since it leads to significant economic losses. One of its main causes is the cross-contamination of food products from industrial surfaces. Three spoilage bacterial species which are highly present in meat and the gastrointestinal tract of chickens were selected: *Pseudomonas fragi*, *Leuconostoc gasicomitatum*, and *Lactobacillus reuteri*. The dual aim was to determine their ability to form monospecies biofilms and to examine how they interact when they coexist together. To do so, mature monospecies biofilms were produced statically for seven days at a temperature of 30 °C. *L. gasicomitatum* was also used to investigate the behavior of *P. fragi* and *L. reuteri* in the formation of multispecies biofilms. The structure and composition of the monospecies biofilms were evaluated by direct epifluorescence microscopy, and the multispecies biofilms were evaluated by plate counting. Both *L. gasicomitatum* and *L. reuteri* were able to form biofilms, with counts of approximately 7 Log CFU/cm^2^ and a defined structure. However, *P. fragi* obtained counts to the order of 4 Log CFU/cm^2^, which is significantly different from the previous species (*P* < 0.05), and it had no network of cell conglomerates. The content of the *L. gasicomitatum* and *L. reuteri* biofilm matrices were 70–80% protein, unlike *P. fragi*, which presented a higher polysaccharide content (*P* < 0.05). In the multispecies biofilms, the presence of *P. fragi* did not affect the growth of *L. gasicomitatum*, which remained at between 5.76 to 6.1 Log CFU/cm^2^. However, *L. reuteri* was able to displace *L. gasicomitatum* growth after 24 h of coexisting in a mixed biofilm, presenting differences in counts of approximately 2 Log CFU/cm^2^. The study of the biofilms constructed by food industry resident microbiota can help to understand the ecological relations that exist between species, characterize them, and propose strategies to eliminate them. The name of genes and species should be written in italic.

## 1. Introduction

Microbial capacity to adhere to industrial surfaces and subsequently initiate biofilm formation has important implications for the food industry, especially in terms of food safety and quality [1]. Biofilms are defined as complex microbiological ecosystems generally made up of multiple associated species which are adhered to a surface and embedded in a protective extracellular matrix [2,3]. This microbial association has been demonstrated to be a form of protection against hostile environmental conditions, and another way to promote symbiotic relationships between species [4,5,6] to favor their persistence, leading to recurrent contamination problems [7]. In the food industry, biofilm presence is considered as undesirable since it increases the risk of microbial cross-contamination to food products and, when pathogens are present, the possibility of foodborne disease transmission [8]. If spoilage microorganisms comprise these microbial communities, cross-contamination can also lead to a decrease in shelf life or a loss of product quality, a factor that is also highly relevant for the food industry and poorly studied by researchers.

The extracellular matrix is a very important component of biofilms and can represent more than 90% of the total mass of these structures [9]. In general, proteins and lipids are among the main molecules that constitute the matrix, exhibiting amyloid-like properties, in addition to exopolysaccharides and eDNA [10]. The microbial cells inside this matrix are fully protected against unfavorable conditions, such as changes in the environment, aggressive chemical and physical agents, antibiotics, and disinfectants [8]. Different advantages have been described for microbial cells when they grow in biofilms, the most important of which are protection against adverse conditions, the increased availability of nutrients for their growth, water availability, a reduced risk of dehydration, and the proximity to other bacteria, facilitating genetic exchange [11]. The matrix must be structured and robust to be able to exercise these functions. In this regard, one of the strategies employed by several bacterial species to make the biofilm structurally rigid is to synthesize protein fibers, which generate a framework onto which cells and other matrix components, such as exopolysaccharides bind [12,13]. Another important aspect of matrices is that their nature depends entirely on the microbiota they are comprised of, so their composition can vary depending on the type of microorganisms that constitute the biofilms [10]. The study of the extracellular matrix produced by spoilage microorganisms may therefore be highly interesting for combating biofilms in the food industry, where cleaning and disinfection operations become key aspects for the control of these structures [8,14]. Generally, the products used to disinfect in the food industry do not penetrate the biofilm matrix, hence this structure protects the microorganisms, which are consequently not eliminated. Furthermore, the cells become resistant when they are exposed to sub-lethal concentrations of disinfectants [15,16]. Therefore, alternative strategies for their control in the food industry are required, such as the use of bacteriophages [17], *quorum sensing* inhibitors [18], or essential oils [19], or through biocontrol using microorganisms that can generate an ecological replacement by competition [20].

The meat industry is the fourth industrial sector in Spain, representing 12% of GDP, and it is the first in the Spanish food and beverage industry, with a turnover of 24,000 million euros, 22.3% of the entire food sector [21]. The contamination of meat products by spoilage microorganisms and the associated loss of quality can, therefore, have a huge economic impact on the meat industry not only at a national level but also at an international one. Meat is susceptible to cross-contamination during slaughter, carnation, processing, and storage operations [22]. Gram-negative bacteria, such as *Pseudomonas* spp., *Enterobacteriaceae*, *Brochothrix thermosphacta*, and Gram-positive bacteria, such as lactic acid bacteria (LAB), dominate among the meat spoilage microbiota [23]. It has been demonstrated that *Pseudomonas fragi*, *Pseudomonas lundensis*, and *Pseudomonas fluorescens* are some of the species of the genus *Pseudomonas* spp. that contribute most to spoilage, and these have been shown to be present in meat processing environments [24]. Furthermore, *P. fragi* is one of the largest producers of ethyl esters, which produce both the sweet, fruity smell characteristic of the initial phase of deterioration, and the putrescine from arginine and a series of sulfur compounds responsible for the putrid odors in the advanced phase of alteration [25,26]. The environment generated with vacuum-packed meats inhibits the growth of the aerobic Gram-negative microbiota and favors the predominance of certain lactic acid bacteria, mainly *Leuconostoc* spp. and *Lactobacillus* spp. [27]. Some of these microorganisms are part of the microbiota of poultry [28], which reach the food industry environment during processing, settling in niches on the surfaces and potentially contributing to cross-contamination. To this effect, *L. gasicomitatum* is of special interest, since it has been repeatedly isolated in the meat industry [29]. Studies have also been carried out to determine the LAB in the intestinal tract of chickens and their subsequent relationship with spoilage, concluding that *L. reuteri* is the most abundant among them [30,31,32].

In the present study, the main objectives were, first, to determine the capacity of the main contributors to meat spoilage, *P. fragi*, *L. reuteri*, and *L. gasicomitatum*, which have in common their association with the meat industry, to form monospecies biofilms; and second, to evaluate the effect of preimplanted biofilms of *L. gasicomitatum* on the survival and viability of *P. fragi* and *L. reuteri*, in addition to the type of interaction exerted.

## 2. Materials and Methods

### 2.1. Surfaces to Test

AISI 316 grade 2B stainless steel coupons, 1 mm thick and 2 cm in diameter, were used to perform the different studies. Cleaning and disinfection processes were applied to the coupons, first by subjecting them to a non-bactericidal detergent (ADIS Higiene, Madrid, Spain) and afterwards to 70% iso-propanol (Panreac, Castellar del Vallès, Spain). The surfaces were then air-dried in a laminar flow cabinet according to protocol UNE-EN 13697 for non-porous materials [33] and further autoclaved for 15 min at 121 °C prior to bacterial inoculation to ensure their complete sterility.

### 2.2. Bacterial Strains

Three spoilage bacteria were used in this study: *Leuconostoc gasicomitatum* CECT 5767, *Pseudomonas fragi* CECT 446, and *Lactobacillus reuteri* CECT 925. The isolates were obtained from the Spanish Type Culture Collection (CECT, Paterna, Spain). The common link between all the strains was their isolation from meat and the gastrointestinal tract of chickens and, therefore, their relationship as spoilage microorganisms in the meat industry [28,30,31,32]. The strains were stored at 4 °C as freeze-dried cultures, recovered on Tryptic Soya Broth (TSB, bioMérieux, Marcy l’Etoile, France) at 30 °C for 48 h, streaked onto Tryptone Soya Agar (TSA, Oxoid, Madrid, Spain), and cultivated at 30 °C for 48 h. Last, the working cultures were kept on TSA slants at 4 °C to be used within 30 days.

### 2.3. Monospecies Biofilms

#### 2.3.1. Inoculum Preparation

The inoculum was prepared with 24 h stationary cultures. Isolated colonies from these cultures were inoculated in 10 mL of TSB for *P. fragi* and *L. gasicomitatum*, and in 10 mL of MRS (Oxoid, Hampshire, England) for *L. reuteri*, until a turbidity of 1.2–1.5 McFarland units was reached. Decimal dilutions in TSB were subsequently made until a concentration of 10^6^ CFU/mL, the established microbial concentration for biofilm formation assays [34], was reached. It has been noted that *L. reuteri* in TSB has a slight growth, so MRS broth was used for biofilm formation as this is specifically for LAB [28].

#### 2.3.2. Biofilm Formation

To produce the bacterial biofilms, 30 µL of the bacterial suspension was inoculated in the center of each stainless steel coupon, resulting in a surface concentration of 5 Log CFU/cm^2^. The coupons were placed in sterile Petri dishes and subsequently inserted into a humidity chamber maintained at saturated relative humidity, and incubated at 30 °C with the objective of promoting biofilm growth under moist conditions [35]. The biofilms were formed over a total incubation period time of one week in static conditions, with a series of washing steps and the drawing of nutrients by adding more culture medium. These steps were followed at 48 h + 24 h + 24 h + 72 h. The culture medium renewal was performed by washing the inoculated coupons twice with 3 mL of sterile distilled water and adding 30 µL of TSB for *P. fragi* and *L. gasicomitatum*, and 30 µL of MRS for *L. reuteri*, to enhance the growth of the attached cells and promote biofilm formation [34]. Last, the stainless steel coupons were once again placed under the established test conditions.

#### 2.3.3. Evaluation of Cell Viability and Matrix Composition of the Biofilms by Direct Epifluorescent Microscopy (DEM)

The stainless steel surfaces were stained with 5 µL of Live/Dead BacLight (Molecular Probes, Eugene, OR, USA) to evaluate cell viability. This kit is composed of two fluorescent dyes of nucleic acids, SYTO9 and propidium iodide (PI). The first penetrates cells with either intact or injured membranes. In contrast, PI penetrates only the injured membrane cells and reduces the SYTO9 dye. Therefore, on applying these two dyes in appropriate proportions the viable cells with intact membranes show up in fluorescent green, and dead, killed, or injured cells show up in fluorescent red. After the staining, the samples were incubated in darkness at 20–22 °C for 15 min according to the manufacturer′s instructions and further analyzed by direct epifluorescent microscopy (DEM).

A mixture of three fluorocroms, Concanavalina A-Alexa Fluor 594 (ConA 594; ThermoFisher Scientific, Barcelona, Spain) which stains in red, Fluorescein-5-isothiocyanate (FITC, Sigma-Aldrich, Madrid, Spain) which stains in green, and 4′,6-diamino-2-phenylindole (DAPI, ThermoFisher Scientific, Barcelona, Spain) which stains in blue, were used to assess the composition of the matrix. To obtain the final staining solution, 1 mg/mL of each of the different fluorocroms were mixed with 0.1 M of sodium bicarbonate (NaHCO_3_, Panreac, Castellar del Vallès, Spain). On each disc, 20 μL of ConA, 10 μL of FITC, and 20 μL of DAPI were added together with 150 μL of 0.1 M NaHCO_3_. Once the 200 μL was deposited on the discs, the samples were incubated in darkness at 20–22 °C for 1 h so that the dyes could penetrate the structure. The samples were subsequently analyzed using DEM.

All the readings were taken with an epifluorescent microscope BX51/BX52 (Olympus, Tokyo, Japan) equipped with a mercury lamp of 100 W (USH-103OL, Olympus), a double pass filter (U-M51004 F/R–V2, Olympus, Tokyo, Japan), and a digital camera (DP50-CU, Olympus). The stained samples were observed with 20× objective. For each sample, six random images were taken from six different fields. The images were analyzed using the analySIS Auto 3.2 software (Soft Imaging System, Münster, Germany).

### 2.4. L. gasicomitatum Preimplantation on Stainless Steel Surfaces and Its Effect on Subsequent Colonization by P. fragi and L. reuteri

#### 2.4.1. Inoculum Preparation

First, a preimplantation of *L. gasicomitatum* was carried out on the study surfaces. To do so, *L. gasicomitatum* was cultivated on TSA at 30 °C for 24 h to achieve stationary phase cultures. Isolated colonies were then introduced into TSB (1.2–1.5 McFarland units), and decimal dilutions were made also in TSB until a concentration of 10^6^ CFU/mL was reached, as described in Section 2.3.1.

#### 2.4.2. *L. gasicomitatum* Preimplantation and Subsequent Colonization

The microorganism was preimplanted on the surface following the same protocol as for the formation of monospecies biofilms. To do so, 50 µL of the bacterial suspension was inoculated in the center of each stainless steel coupon. The inoculated surfaces were incubated, washed, and renewed for nutrients following the same procedure as established in Section 2.4.2., with the only difference that 50 μL of the sterile TSB medium was added as nutritive replacement. After seven days of *L. gasicomitatum* biofilm formation, the two other bacterial strains, *P. fragi* and *L. reuteri*, were inoculated on the preimplanted structure. For this, both *P. fragi* and *L. reuteri* were cultivated in TSA and incubated at 30 °C for 24 h. The bacterial inoculums were prepared following the same procedure as in Section 2.4.1. until a concentration of 10^6^ CFU/mL was reached, at which point 30 µL were inoculated on the preimplanted biofilms of the *L. gasicomitatum* strain. The surfaces were incubated in a humid chamber at 30 °C for 24, 48, and 72 h. The samples were evaluated after these various hours of incubation to obtain a response on the interaction exerted.

#### 2.4.3. Multispecies Biofilm Evaluation by Plate Count

Plate count was established as the methodology to determine the bacterial growth of each of the strains in multispecies biofilms after incubation periods of 24, 48, and 72 h. To do so, the surfaces were washed twice with 3 mL of sterile distilled water to remove the unattached cells and then placed in a sterile flask containing 3.5 g of glass beads and 10 mL of peptone water. The samples were then vortexed for 90 s at 40 Hz to dislodge the attached cells from the surface for quantification [36].

The resulting suspension was decimally diluted in peptone water and transferred to a plate for its quantification. Since the biofilms consisted of two species, a culture medium was designed that enabled them to be differentiated. The media consisted of esculin, since *L. gasicomitatum* was observed to ferment the sugar, while *P. fragi* and *L. reuteri* did not. This enabled a medium composed of TSA, esculin (Sigma-Aldrich, Madrid, Spain), and iron citrate (Sigma-Aldrich, Madrid, Spain) to be developed, which turned the colonies of *L. gasicomitatum* black, making it easily distinguishable from the other two strains used. Differences were observed based on colony morphology. The plates were incubated at 30 °C for 48 h and then counted.

### 2.5. Statistical Analysis

All the tests were performed in duplicate on three independent days (*n* = 6). The bacterial counts were converted into decimal logarithmic values to almost match the assumption of a normal distribution. The results were evaluated using an analysis of variance (ANOVA) with a posteriori contrast using the Tukey test. The statistical software package SPSS Statistics IBM (Armonk, NY, USA) 23 was used throughout. A *P* < 0.05 was considered as statistically significant. The statistical analysis of the variance was used to compare the three different strains used in the studies, including monospecies and multispecies biofilms.

## 3. Results and Discussion

### 3.1. Evaluation of the Formation Capacity of the Monospecies Biofilms

The main objective of this study was to know the biofilm formation capacity of *P. fragi*, *L. reuteri*, and *L. gasicomitatum*, by quantifying the viable and non-viable cells, and by observing the generated structure and established cellular organization. This evaluation was considered as important since not all microorganisms are capable of forming biofilms on stainless steel surfaces, or of forming them with the same intensity. *Campylobacter* spp., for example, does not usually form own biofilms but manages to persist in the food industry by invading the biofilms formed by other microorganisms [37]. The aim was to establish whether these microorganisms can form biofilms and persist under industrial conditions, producing cross-contamination to food products, if they end up on stainless steel surfaces.

The three bacteria used in the study, *P. fragi*, *L. reuteri*, and *L. gasicomitatum*, proved to be capable of adhering to the stainless steel surfaces, grow and develop cellular structures, but at different intensities (Table 1). As can be observed, the total cell count that conformed the biofilms of *L. reuteri* and *L. gasicomitatum* differed significantly (*P* < 0.05) from the other species under study. This result could be due to *P. fragi* not adhering strongly enough to the surface, leading to the non-adhered cells being discarded when performing the washes and a part of the biofilm structure to be lost along with the cells. It is important to consider that the properties of the different surfaces used in the food industry differ among them, directly influencing microbial adhesion and subsequent biofilm formation [38,39]. In this regard, *Pseudomonas* spp. could have a greater affinity to adhere to other types of surfaces, such as plastics [40].

As can also be observed in Table 1, the survival percentage of the biofilms generated by the three bacterial species was also measured by calculating the relation between non-viable cell count with respect of total cell counts, all of which formed part of the structure produced. The resulting viable cell percentage ranged between 0.03% and 10.34%, the highest percentage corresponding to *L. reuteri* biofilms. Nonetheless, no significant differences (*P* > 0.05) were found between any of the species. The fact that the non-viable cell count was high could be due to various reasons. One of them could be that with long incubation times the bacteria that constitute the biofilm exceed their exponential growth curve, overcoming the stationary phase and causing cell death, helping to give the system structure and providing the cells that remain viable in the biofilm with a new source of energy [34]. This has been observed in the study of biofilms of other microorganisms, such as *Bacillus subtilis*, in whose non-viable cells complex three-dimensional structures are generated, constituting a stress response at the community level to improve the biofilm′s resistance to unfavorable environmental conditions [41]. Another explanation for the high number of non-viable cells compared to the viable cells could be the analytical technique used. The depth of the structure is not considered when using DEM as a methodology for biofilm formation, since this analytical technique only provides a 2D image. Biofilms, however, are 3D structures, so to be able to evaluate the presence of viable cells inside the biofilm other techniques are required, such as confocal laser microscopy, which allows reconstructions and three-dimensional analyses of the acquired images to be made [42]. In this sense, the non-viable cells are preferably located in the outermost layers of the biofilm, and do not consume substrate, allowing it to penetrate inside the structure to feed the innermost cell layers, and exerting a certain protective role against possible antimicrobial agents [43]. Hence, there are various reasons why the viable cell count could have been underestimated.

**Figure 1:**
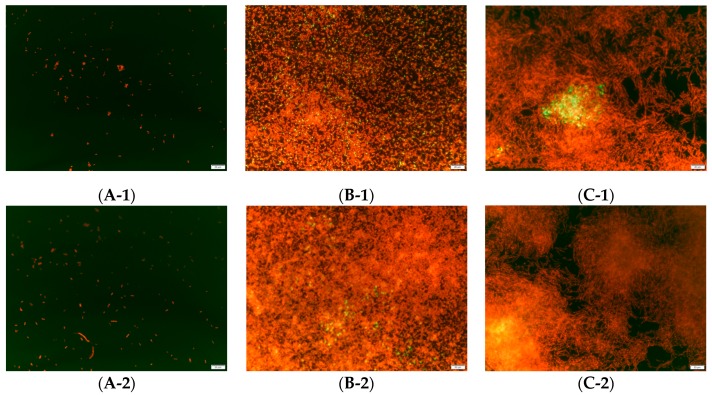
Images obtained by direct epifluorescent microscopy (DEM) for the quantification of viable and non-viable cells using Live/Dead BacLight biofilm stain for: (**A-1**;**A-2**) *P. fragi*; (**B-1**;**B-2**) *L. reuteri*; (**C-1**;**C-2**) *L. gasicomitatum*. Magnification 20×.

**Figure 2:**
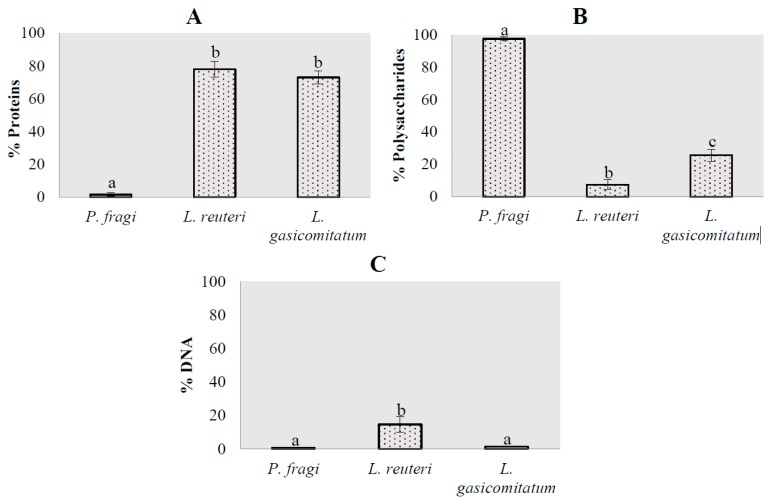
Representation of the percentages of the macrocomponents that form the biofilm matrices of the different bacterial species under study: (**A**) proteins; (**B**) polysaccharides; (**C**) DNA. Error bars indicate the standard error of the mean. Columns lacking a common letter differ significantly (*P* < 0.05).

Biofilm formation can also be determined by the organization of the cells that form it, observed by DEM [36]. Accordingly, an arrangement of disaggregated cells indicates that biofilms have not formed, while the presence of cells that are beginning to aggregate and form a three-dimensional network signifies that a biofilm with an organized and compact structure has been established on the surface [44]. This is considered as an important observational measure, since obtaining a count of cells adhered to the surface does not necessarily imply that the microorganism has triggered the formation of biofilms. This point is demonstrated in Figure 1 (A-1, A-2), which corresponds to *P. fragi*. A total count of 4.82 Log CFU/cm^2^ was obtained for this bacterium, but no connected network between cells was observed; in fact, the cells were completely dispersed on the surface. Therefore, it can be assumed that under the experimental conditions tested, *P. fragi* did not have the capacity to form biofilms. *L. reuteri* and *L. gasicomitatum*, however, presented the opposite behavior. Both microorganisms were shown to have counts in the order of 7 Log CFU/cm^2^, with no significant differences between them (*P* > 0.05). Unlike *P. fragi*, these cells could have adhered more strongly to the surface, thus resisting washes. The objective of the washes was always to discard the cells not adhered to the surface, and which were, therefore, not part of the structure. However, if the biofilm formation capacity is weak, this can be a determinant for releasing biofilm structures under production. This was not observed in *L. reuteri* and *L. gasicomitatum*, since both species were shown to have a high biofilm formation capacity by presenting a complex and highly ordered structure, as shown in Figure 1B-1,B-2 and Figure 1C-1,C-2, including cellular conglomerates with interstitial voids indicative of mature biofilms [34]. It has been suggested that empty areas within the structure (i.e., interstitial voids) may be water channels, which promote the constant circulation of nutrients and the elimination of waste [34,45,46].

Last, interesting to note was the yellow color observed in certain areas (Figure 1B,C) of the cellular structures generated by *L. reuteri* and *L. gasicomitatum* for the biofilm formation. This color may be produced by cell lysis with the consequent release of e-DNA [47]. However, it could also be caused by the superposition of viable and non-viable cells (i.e., green and red, respectively) which, when mixed together, would result in the yellow color. This would again indicate that the structure harbors viable cells in deeper areas [48], although further studies would be needed to prove this.

### 3.2. Matrix Composition in Monospecies Biofilms

Production of the biofilm matrix, which encompasses and structures the biofilm, was evaluated by DEM for the three bacterial species. The study of this process presents a huge challenge due to the large amount and heterogeneity of the biopolymers and other substances involved [8,12,49]. However, the qualitative and quantitative evaluation of this production is of huge interest, since the results can be an advance for developing products for their elimination.

The results obtained for the macromolecule composition of the formed biofilms at a quantitative level are shown in Figure 2. *L. reuteri* and *L. gasicomitatum* were the species that presented the highest protein content percentages (Figure 2A), with no significant differences (*P* = 0.605) between them, unlike for *P. fragi* (*P* < 0.05). These results were in accordance with Combrouse et al. [50] and Colagiorgi et al. [51], which demonstrated that the matrix produced by *Listeria monocytogenes* is mostly composed of protein. Which compounds make up the extracellular matrix of microbial biofilms, established as mainly polysaccharides, has been a subject of controversy in recent years. Several studies have concluded that the nature of the matrix produced is dependent on the bacterial species [34,49], which was observed in this study. Contrarily, *P. fragi* showed a predominance of polysaccharides as a structural compound in the biofilms (Figure 2B), presenting significant differences (*P* < 0.05) compared to the other two species. The results obtained are in accordance with other studies on the matrix produced by *Pseudomonas* spp., which conclude that most of them are formed by hydrocarbon compounds, especially alginate [52]. All this points to the nature of the matrix produced being related to the characteristics of the cell wall. In this regard, it has been shown that Gram negative bacteria, such as *Salmonella* spp., *Acetobacter xylinum*, and *Legionella pneumophila*, produce biofilms mostly with a polysaccharide composition [52,53,54], similar to the results obtained for *P. fragi*. Differently, in Gram positive bacteria for *L. monocytogenes*, the matrix was mainly produced from protein content, as described by Colagiorgi et al. [51] and as was observed for *L. reuteri* and *L. gasicomitatum* in the present study.

Last, the percentage of e-DNA was determined to be an integral part of the matrix composition of bacterial biofilms (Figure 2C). No significant differences (*P* = 0.983) were found between *P. fragi* and *L. gasicomitatum*, while *L. reuteri* presented with *P. fragi* (*P* = 0.001) and *L. gasicomitatum* (*P* = 0.002), the latter having the highest percentage. It has been observed that e-DNA not only plays a structural role in microbial biofilms, but it also serves as a source of energy and nutrients [10,55]. The latter could be one of the reasons why biofilms have such a low proportion of this compound.

**Figure 3:**
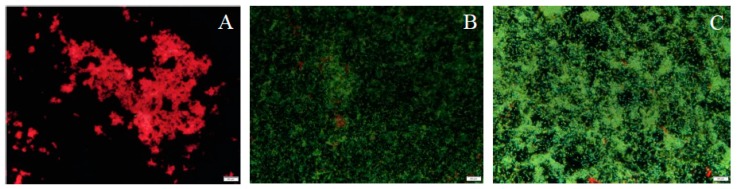
Images obtained by DEM for the quantification of the macromolecules that form the biofilm extracellular matrix stained with Fluorescein-5-isothiocyanate (FITC) in green, ConA in red, and 4′,6-diamino-2-phenylindole (DAPI in blue, and visualized by DEM with 20× for: (**A**) *P. fragi*; (**B**) *L. reuteri*; (**C**) *L. gasicomitatum*. Magnification 20×.

At a qualitative level and unlike the results obtained in the cell viability study, it was observed that *P. fragi* presented some areas with organized and defined structures (Figure 3A). In accordance with the results of the quantitative study, these were composed of polysaccharides. This result is noteworthy since no extracellular matrix was expected to form, given that it did not demonstrate the ability to produce cell conglomerates and thus biofilms. Although there was no biofilm formation, it can be said that there was some cellular activity when the production of these compounds was observed. For both *L. reuteri* and *L. gasicomitatum* (Figure 3B,C), proteins clearly dominated in the biofilm mass, minimally mixed with some glycidic components, among which were glycoproteins and mucopolysaccharides [34]. However, there seemed to be a dominance of the presence of proteins on the surface. e-DNA appears as a minor component dispersed throughout the matrix. It is interesting to note that the results obtained in this study could be used for developing specific, effective products to be applied as new cleaning and disinfection strategies based on a clearer understanding of the main components to be attacked in each species.

### 3.3. Preimplantation of L. gasicomitatum and Influence on the Growth of P. fragi and L. reuteri

The microbial communities that constitute biofilms can be composed of one or multiple species, although the latter predominates in the food industry [8,56]. There are few studies that attempt to recreate a multispecies microbial community to observe how different microorganisms interact with each other, since these procedures are difficult to perform [29]. The main objective of this study was to evaluate the effect that preformed biofilms of *L. gasicomitatum* have on the two remaining bacterial species under study, *P. fragi* and *L. reuteri*, to observe their influence on subsequent adhesion and growth. *L. gasicomitatum* was selected as the base microorganism because in previous studies on the isolation of microbiota resident in an Iberian pig processing industry carried out by Ripolles-Avila et al. [20] and Hascoët et al. [29], *Leuconostoc* spp. was found to be one of the most predominant genera within the microorganisms isolated, revealing their potential importance in product cross-contamination. This concurs with other studies in which *Leuconostoc* spp. have been highly detected both in sausage processing environments and in the final product, suggesting the possible existence of microbial reservoirs on food contact and industrial surfaces [57,58]. Furthermore, *L. gasicomitatum* could generate mature biofilms with other microorganisms adhering to it when they remain on surfaces. The aim, therefore, was to investigate whether *P. fragi* and *L. reuteri* can survive and even grow in the structure formed by *L. gasicomitatum*, serving as a support and protection.

The results obtained for the biofilms formed by *L. gasicomitatum* and *P. fragi* are shown in Table 2. In previous experimental tests, it has been observed that even when *P. fragi* presents a certain cell count on the surface, it is not able to generate a mature biofilm. Therefore, it has been suggested that this microorganism has very low adhesion strength on stainless steel surfaces, leading to its loss. However, in the present experimental study *P. fragi* was observed to coexist and even grow exponentially in a multispecies community with *L. gasicomitatum* during the first 48 h. This fact has also been verified for other species of *Pseudomonas* spp., such as *Pseudomonas putida*, when grown in mixed biofilms with *Acinetobacter* spp., where it was observed that the two species in coexistence generated a more complex biomass and increased *P. putida* counts at the expense of *Acinetobacter* spp., the number of cells of which may have slightly decreased due to limited access to oxygen [59]. Thus, it is vital to investigate the behavior of bacterial species in both monospecies and multispecies biofilms to determine when the biomass increases and the general function of the microbial community, and to understand the type of interaction these bacterial species generate in the system at a cooperative, synergistic, and competitive level [60,61]. After an incubation period of 72 h, the population of *P. fragi* reduced, possibly due to competition for nutrients which would have begun to be scarce, given that they were not replenished. These results concur with Flemming et al. [6] and Iñiguez-Moreno et al., [62], which indicated that nutrient depletion causes increased competition and, therefore, cell death. Another noteworthy result is that the counts of *L. gasicomitatum* remained the same after inoculating *P. fragi* into the preimplanted biofilms of *L. gasicomitatum* and an incubation period of 24 h, coinciding fully with the control and without presenting significant differences (*P* > 0.05) in any of the incubation periods. *P. fragi*, however, presented differences throughout the entire process when coexisting in multispecies biofilms (*P* < 0.05), which is why it can be suggested that *L. gasicomitatum* growth is not affected by the presence of *P. fragi* in the environment. The results obtained by DEM in the study of monospecies biofilms of *P. fragi* (total cell count 4.82 Log CFU/cm^2^, and survival rate 0.03%) were similar to those obtained when it was cultivated as a multispecies biofilm with *L. gasicomitatum*. However, the total count values obtained by DEM mostly corresponded to non-viable cells, as opposed to the counts obtained in the mixed biofilm, which represented the number of cells in a viable state since plate count was used as an analytical technique. Hence, it may be indicated that *P. fragi* is compatible with *L. gasicomitatum*. Despite being able to reconcile its growth requirements, and as discussed above, *P. fragi* counts decreased after the maximum incubation time of 72 h, although without presenting statistically significant differences (*P* > 0.05) from the previous incubation times. Further studies are required to determine if *P. fragi* counts would continue to decrease with increased incubation times as indicated by the trend, in addition to studies of the structure generated in multispecies biofilms to understand if *P. fragi* is included within the structure, allowing this bacteria to adhere and develop adequately.

The interaction observed between *L. gasicomitatum* and *L. reuteri* was different from that observed in the previous case (Table 3). After 24 h of incubation, the *L. gasicomitatum* population decreased by approximately 2 Log (CFU/cm^2^) with respect to the control, but without any statistically significant differences (*P* > 0.05). This could be attributed to variability, so a larger number of samples would be needed to determine whether the initial effect is real. After 24 h of incubation, the microbial *L. gasicomitatum* curve coincided with the presented biofilm monospecies curve, with no significant differences (*P* > 0.05). In this case, the same reported trend can be observed as for multispecies biofilms between *L. gasicomitatum* and *P. fragi*: the *L. reuteri* population decreased, this time significantly (*P* < 0.05). This could be due to a decrease in nutrients [6], as previously discussed above.

## 4. Conclusions

The study of monospecies and multispecies biofilms in vitro is of enormous interest for the food industry to understand how they behave and to find ways to eliminate them. Based on the results obtained in the present study, it can be concluded that *P. fragi* is not able to form biofilms under the established experimental conditions, adhering weakly and in a dispersed way. Contrarily, *L. reuteri* and *L. gasicomitatum* demonstrated the ability to form biofilms with high cell density, giving rise to a structure with a complex, mature network. Regarding the composition of the macromolecules in the matrix, *L. reuteri* and *L. gasicomitatum* had a higher percentage of proteins, while the majority compound of *P. fragi* was polysaccharides, although their high presence could be attributed to the wall compounds of the bacterial cell. The minor component found was e-DNA, except for *L. reuteri*. In addition, both *P. fragi* and *L. reuteri* can survive and develop within the structure generated in the pre-implanted biofilm of *L. gasicomitatum*, although at a different level compared to in monospecies biofilms. The growth of *L. gasicomitatum* in mature biofilms was not affected by the presence of *P. fragi* in any of the set incubation times. However, *L. reuteri* was shown to have some effect on the displacement of *L. gasicomitatum* after 24 h in coexistence as a mixed biofilm. Nonetheless, further studies are needed to corroborate this effect. Finally, both *P. fragi* and *L. reuteri* demonstrated a population decline after 48 h of coexistence in a mixed biofilm with *L. gasicomitatum*. It was determined that this could be due to a decrease in nutrient availability, leading to competition and, consequently, cell death.

## Figures and Tables

**Table 1:** Total counts and survival percentage of the cells forming the biofilms for the different bacterial species.

Microorganism	Total Count (Log CFU/cm^2^)	% Survival *
*P. fragi*	4.82 ± 0.12 ^a^	0.03 ± 0.02 ^a^
*L. reuteri*	7.10 ± 0.05 ^b^	10.35 ± 5.85 ^a^
*L. gasicomitatum*	7.05 ± 0.26 ^b^	0.92 ± 0.87 ^a^

Each value corresponds to an average of two repetitions performed on three separate days (*n* = 6). Standard error of the mean was included. * Survival percentage calculated by obtaining the relation between non-viable cell count with respect of total cell counts. ^a,b^ Values within a column lacking a common superscript differ significantly (*P* < 0.05).

**Table 2:** Quantification of the cells forming the multispecies biofilms between *L. gasicomitatum* and *P. fragi* at 24, 48, and 72 h of incubation.

Bacteria	Incubation Period as Multispecies Biofilm (Hours)		
	24	48	72
*P. fragi*	3.69 ± 0.66 ^aA^	4.73 ± 0.33 ^aA^	4.46 ± 0.29 ^aA^
*L. gasicomitatum*	5.98 ± 0.22 ^bA^	6.10 ± 0.18 ^bA^	5.76 ± 0.22 ^bA^
Control *L. gasicomitatum*	5.73 ± 0.16 ^bA^	6.17 ± 0.15 ^bA^	5.76 ± 0.20 ^bA^

Each value corresponds to an average of two repetitions performed on three separate days (*n* = 6). Standard error of the mean was calculated. ^a,b^ Values within a column lacking a common lowercase letter differ significantly (*P* < 0.05). ^A,B^ Values within a row lacking a common capital letter differ significantly (*P* < 0.05).

**Table 3:** Quantification of cells forming the multispecies biofilms between *L. gasicomitatum* and *L. reuteri* at 24, 48, and 72 h of incubation.

Bacteria	Incubation Period as Multispecies Biofilm (Hours)		
	24	48	72
*L. reuteri*	5.42 ± 0.73 ^aA^	6.21 ± 0.17 ^aA^	4.63 ± 0.64 ^aA^
*L. gasicomitatum*	5.00 ± 0.67 ^aA^	6.32 ± 0.04 ^aA^	5.97 ± 0.13 ^bA^
Control *L. gasicomitatum*	6.72 ± 0.03 ^aA^	6.36 ± 0.09 ^aB^	6.03 ± 0.12 ^bB^

Each value corresponds to an average of two repetitions performed on three separate days (*n* = 6). Standard error of the mean was calculated. ^a,b^ Values within a column lacking a common lowercase letter differ significantly (*P* < 0.05). ^A,B^ Values within a row lacking a common capital letter differ significantly (*P* < 0.05).
